# Downregulation of miR-1826 Indicates a Poor Prognosis for Osteosarcoma Patients and Regulates Tumor Cell Proliferation, Migration, and Invasion

**DOI:** 10.1155/2020/7968407

**Published:** 2020-02-11

**Authors:** Peng Li, Lei Wei, Wenshuai Zhu

**Affiliations:** ^1^Department of Spine Orthopaedics, Weifang Traditional Chinese Hospital, Weifang, Shandong 261041, China; ^2^Department of Spinal Surgery, Weifang People's Hospital, Weifang, Shandong 261041, China; ^3^Department of Orthopedics, Weifang People's Hospital, Weifang, Shandong 261041, China

## Abstract

**Background:**

Osteosarcoma (OS) is the most frequent bone tumor with high metastasis. This study is aimed at assessing the expression and prognostic significance of microRNA-1826 (miR-1826) in OS patients, as well as its biological function in tumor progression.

**Methods:**

Quantitative Real-Time PCR was employed to measure the expression of miR-1826 in OS tissues and cell lines. Kaplan-Meier survival analysis and Cox regression model were used to evaluate the prognostic value of miR-1826. CCK-8 and Transwell assay were conducted to investigate the effect of miR-1826 on OS cell proliferation, migration, and invasion.

**Results:**

miR-1826 expression was downregulated in OS tissues and cell lines and associated with OS patients' clinical stage and distant metastasis. Low levels of miR-1826 were related with shorter survival time and determined as an independent prognostic indicator for the overall survival of OS patients. The overexpression of miR-1826 in OS cells led to inhibited cell proliferation, migration, and invasion.

**Conclusion:**

The decreased expression of miR-1826 predicts a poor prognosis in OS patients, and its overexpression inhibits OS cell proliferation, migration, and invasion. This newly identified miR-1826 provides a novel sight into the pathogenesis of OS and offers a candidate prognostic biomarker and therapeutic target for OS treatment.

## 1. Introduction

Osteosarcoma (OS) is the most frequent primary malignant bone tumor and predominantly occurs among children and adolescents aged 10-25 years, accounting for approximately 3.4% of all childhood tumors [1]. Patients with OS suffer from pain and are prone to fracture, leading to the extremely reduced quality of life [2]. It is reported that OS cells are highly metastatic, which makes it difficult to cure and contributes to poor prognosis [3]. Although there are advances in therapeutic methods, such as surgery, chemotherapy, and radiotherapy, the elusive complex pathological mechanisms of OS remain the major obstacles for the improvement of OS treatment [4]. Therefore, exploration of functional molecules that may be involved in the progression of OS is of great importance for patients with OS.

MicroRNAs (miRNAs) are a group of small noncoding RNA molecules with regulatory function in gene expression at the posttranscriptional level [5]. Moreover, miRNAs also play important roles in the regulation of biological processes, such as cell proliferation, migration, invasion, cell cycle, and cell apoptosis [6]. Numerous miRNAs have been identified and investigated in different types of human disease [7]. In recent decades, the pivotal roles of miRNAs have attracted increasing attention for their clinical significance and biological function in the development of various human cancers [8, 9]. Aberrant expression of diverse miRNAs in tumor samples has been determined as an efficient diagnostic and prognostic biomarker in human malignancies, including OS [10]. In addition, some functional miRNAs have been indicated as potential targets in tumor targeted therapy through modulating oncogenes or tumor suppressor genes [11, 12]. Thus, we believed that the investigation of miRNAs for their clinical significance and biological function could improve the prognosis and treatment of OS.

MicroRNA-1826 (miR-1826) has been studied in several cancers. In human melanoma and colorectal carcinoma, the deregulated miR-1826 has been identified as a biomarker for disease diagnosis and prognosis [13, 14]. In renal cancer, miR-1826 played a tumor suppressor role by inhibiting tumor cell proliferation, migration, and invasion [15]. According to a study by Kobayashi et al., miR-1826 was found to be expressed in OS samples and the inhibition of miR-1826 might contribute to OS cell viability by examining the ATP production [16]. However, the expression patterns and function of miR-1826 in OS progression are still unclear. Therefore, this study is aimed at analyzing the expression of miR-1826 in OS tissues and cells and at further exploring the prognostic value of miR-1826 in OS patients, as well as its regulatory effect on OS cell proliferation, migration, and invasion.

## 2. Materials and Methods

### 2.1. Patients and Sample Collection

This study was designed and performed with the approval from the Ethics Committee of Weifang Traditional Chinese Hospital. A total of 122 OS patients were recruited, who were pathologically diagnosed with OS and underwent surgery in Weifang Traditional Chinese Hospital between 2009 and 2013. None of the patients had received any antitumor therapy before the sampling. One hundred and twenty-two paired tumorous tissues and adjacent nontumorous tissues were collected during the surgery and immediately frozen in liquid nitrogen for further analysis. The demographic and clinicopathological characteristics of the patients are recorded and summarized in Table 1. In addition, the patients were enrolled in a 5-year follow-up survey after the treatment, and we tracked their survival information by telephone. An informed consent was obtained from each patient.

### 2.2. Cell Culture and Transfection

The human osteoblast HFOB1.19 cell line and three OS cell lines HOS, U2OS, and SaOS2 were obtained from the Cell Bank of Type Culture Collection of Chinese Academy of Sciences (Shanghai, China). HFOB1.19 cells were cultured in Ham's F12/DMEM supplemented with 10% fetal bovine serum (FBS; Gibco, Grand Island, NY, USA), and the OS cells were cultured in DMEM added with 10% FBS at 37°C in a humidified CO_2_ (5%) atmosphere. miR-1826 mimic, miR-1826 inhibitor, and the corresponding negative controls (mimic NC and inhibitor NC) were synthesized in GenePharma (Shanghai, China). For cell transfection, U2OS and SaOS2 cells were transfected by Lipofectamine 3000 (Invitrogen, Carlsbad, CA, USA) with the vectors above according to the manufacturer's instruction. The subsequent cell experiments were conducted at 48 h after the transfection.

### 2.3. RNA Extraction

Total RNA was extracted from the tissues and cells by TRIzol reagent (Invitrogen, Carlsbad, CA, USA) following the manufacturer's protocols. The concentration and purity of the RNA was evaluated using NanoDrop 2000 (Thermo Fisher Scientific, Waltham, MA, USA). Afterward, the single-stranded cDNA was synthesized from the RNA by the PrimeScript RT Reagent Kit (Takara, Dalian, China) and stored at -20°C.

### 2.4. Quantitative Real-Time PCR (qRT-PCR)

The relative expression of miR-1826 was measured using qRT-PCR, which was completed using the SYBR Green I Master Mix kit (Invitrogen, Carlsbad, CA, USA) and the 7500 Real-Time PCR System (Applied Biosystems, USA). The final expression value was normalized to U6, and the expression fold change was computed by the 2^−*ΔΔ*Ct^ method.

### 2.5. CCK-8 Assay

A Cell Counting Kit-8 (CCK-8; Beyotime, Nantong, China) was used to analyze the proliferation of OS cells. 48 h after the cell transfection, the OS cells (density of 3 × 10^3^) were seeded into 96-well plates and CCK-8 solution was added into the cells at the time points of 0, 24, 48, and 72 h following a 2 h incubation. The absorbance at 450 nm was estimated using a microplate reader (Bio-Rad, Carlsbad, CA, USA) to reflect the cell proliferation ability.

### 2.6. Transwell Assay

The OS cells with a density of 2 × 10^5^ were seeded into the upper chambers of the Transwell chamber (Corning, USA), which was used to evaluate the abilities of cell migration and invasion. The upper chambers included serum-free culture medium, while the lower chambers were filled with medium added with 10% FBS. In addition, the chambers used for invasion assay were precoated with Matrigel (Corning, USA). After 24 h of incubation at 37°C, the cells in the lower chambers were stained with 0.1% crystal violet (Beyotime, Nantong, China) and counted by an optical microscope (Olympus Corporation, Tokyo, Japan).

### 2.7. Statistical Analysis

All the statistical analyses were performed using SPSS 21.0 software (SPSS Inc., Chicago, IL) and GraphPad Prism 7.0 software (GraphPad Software, Inc., USA). Data obtained from the analyses were presented as mean ± SD. The comparison analysis was conducted using Student's *t*-test or one-way ANOVA. The Kaplan-Meier method and log-rank test were used to perform the survival analysis. Cox regression analysis was applied to evaluate the prognostic independency of miR-1826 in OS patients. The difference with a *P* < 0.05 was considered statistically significant.

## 3. Results

### 3.1. Downregulation of miR-1826 in OS Tissues and Cell Lines

**Figure 1:**
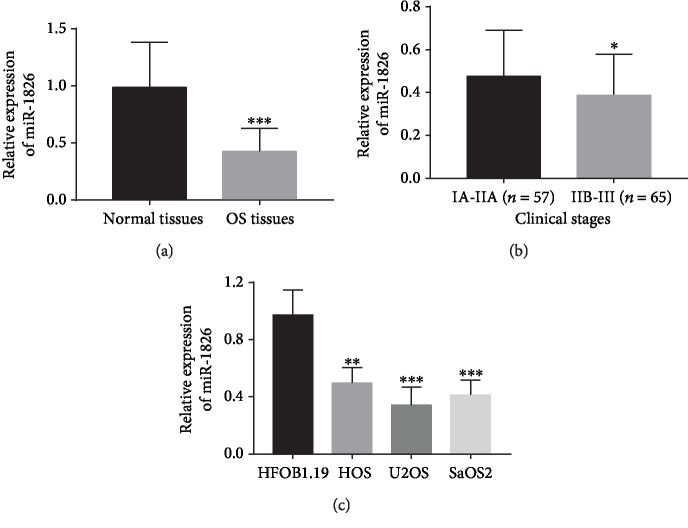
Expression of miR-1826 in OS tissues and cell lines. (a) miR-1826 expression was decreased in OS tissues compared with the normal controls. (b) miR-1826 expression was downregulated in OS patients with advanced clinical stage compared with those with early stage. (c) Downregulated expression of miR-1826 was found in the OS cell lines compared with the normal osteoblast cell line. ^∗^*P* < 0.05, ^∗∗^*P* < 0.01, ^∗∗∗^*P* < 0.001.

By investigating the expression of miR-1826 at the OS patients, we found that the expression of miR-1826 was significantly decreased in the tumor tissues compared with the adjacent normal tissues (*P* < 0.001, Figure 1(a)). Moreover, the 122 OS patients were divided into IA-IIA stage (*n* = 57) and IIB-III stage (*n* = 65) according to the Enneking-Musculoskeletal Tumour Staging System, and the expression of miR-1826 was found to be downregulated in the IIB-III stage patients compared with the IA-IIA stage patients (*P* < 0.05, Figure 1(b)). In addition, the expression data of miR-1826 in OS cells was consistent with the tissue results, which also presented the downregulated expression in OS cells when compared to the normal cells (all *P* < 0.01, Figure 1(c)).

### 3.2. Relationship of miR-1826 Expression with the Clinicopathological Characteristics of OS Patients

Considering the reduction in miR-1826 expression, this study further analyzed the relationship between the miR-1826 expression and the clinical data of the patients. In this analysis, the expression of miR-1826 was classified into low and high levels based on its mean value. As shown in Table 1, we found that the tissue expression of miR-1826 was associated with the patients' clinical stage (*P* = 0.022) and distant metastasis (*P* = 0.026), while its association with age, gender, and tumor size was not obtained (all *P* > 0.05).

### 3.3. Association of miR-1826 Expression with the Overall Survival of OS Patients

**Figure 2:**
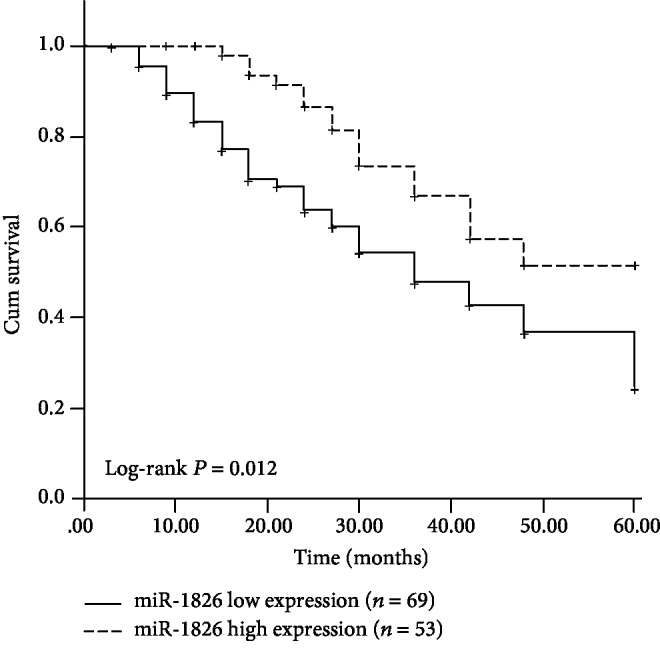
Kaplan-Meier survival curves constructed based on the survival information of OS patients. Low miR-1826 levels were associated with a shorter survival time in OS patients. Log-rank *P* = 0.012.

By using the 5-year follow-up survival information, the Kaplan-Meier survival curves are plotted and shown in Figure 2. Compared with the OS patients with high miR-1826 expression levels, we observed a shorter survival time in the patients with low miR-1826 expression levels (log-rank *P* = 0.012), indicating that the reduced miR-1826 expression might be associated with poor overall survival.

### 3.4. Downregulation of miR-1826 Is an Indicator of Poor Prognosis in OS Patients

To further confirm the prognostic value of miR-1826 in OS patients, a Cox regression analysis that included the clinical features in the equation was performed. From Table 2, the univariate Cox analysis showed that miR-1826, distant metastasis, and clinical stage were associated with the prognosis of OS patients (all *P* < 0.05). The multivariate analysis indicated that miR-1826 was an independent prognostic indicator for the overall survival of patients with OS (HR = 2.452, 95%CI = 1.248‐4.373, *P* = 0.014).

### 3.5. Regulatory Effect of miR-1826 on OS Cell Proliferation, Migration, and Invasion

**Figure 3:**
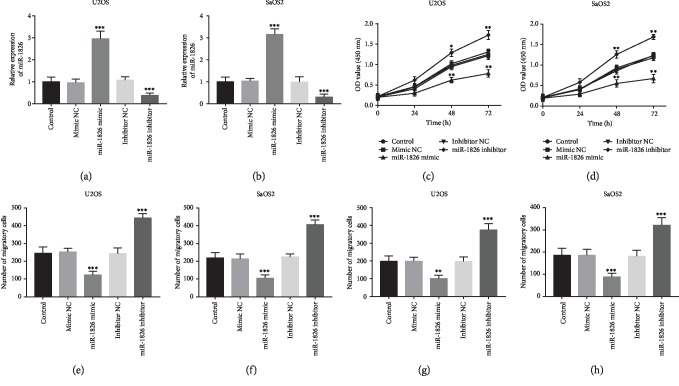
Effect of miR-1826 on OS cell proliferation, migration, and invasion in U2OS and SaOS2 cells. (a, b) The expression of miR-1826 was upregulated by the miR-1826 mimic but was downregulated by the miR-1826 inhibitor. (c, d) The CCK-8 assay indicated that OS cell proliferation was enhanced by the knockdown of miR-1826 but was inhibited by the overexpression of miR-1826. (e–h) Cell migration and invasion abilities measured by Transwell assay were facilitated by the downregulation of miR-1826 but were suppressed by the upregulation of miR-1826. ^∗^*P* < 0.05, ^∗∗^*P* < 0.01, ^∗∗∗^*P* < 0.001.

Given the dysregulation of miR-1826 in OS patients, the biological function of miR-1826 in OS progression was further explored using the gain- and loss-of-function experiments. By cell transfection, the expression of miR-1826 was upregulated by the miR-1826 mimic, while it was downregulated by the miR-1826 inhibitor in U2OS and SaOS2 cells (all *P* < 0.001, Figures 3(a) and 3(b)). By CCK-8 assay, we found that the OS cell proliferation was significantly enhanced by the downregulation of miR-1826 but was inhibited by the upregulation of miR-1826 (all *P* < 0.05, Figures 3(c) and 3(d)). For the migration and invasion assay, the overexpression of miR-1826 in OS cells led to the suppressed migration and invasion abilities, whereas the knockdown of miR-1826 resulted in the increases in cell migration and invasion (all *P* < 0.05, Figures 3(e)–3(h)).

## 4. Discussion

OS is the most frequent primary bone malignancy with a high rate of metastasis and poor prognosis. Novel molecules to accurately predict the prognosis and improve the therapeutic strategies are urgently needed. In this study, the expression of miR-1826 was downregulated in OS tissues and cell lines, and the downregulation of miR-1826 was associated with the patients' clinical stage and distant metastasis. More importantly, the patients with low miR-1826 levels showed poor overall survival compared with those with high miR-1826 levels, and the decreased expression of miR-1826 was determined as an independent indicator for poor prognosis of OS. By cell experiments, the overexpression of miR-1826 was found to inhibit the cell proliferation, migration, and invasion of OS cells. Inversely, the knockdown of miR-1826 led to the opposite effects.

Numerous miRNAs have been investigated in various human diseases, especially in human malignancies [17]. The miRNAs with deregulated expression values play important roles in tumorigenesis. For example, increased expression of miR-421 was observed in non-small-cell lung cancer patients and demonstrated to serve as a prognostic biomarker and tumor progression regulator [18]. Downregulated expression of miR-383-5p was detected in gastric cancer patients, which was related with a shorter survival time, tumor size, and differentiation grades and determined as a tumor suppressor [19]. Zhang et al. investigated the role of miR-942 in hepatocellular carcinoma and gave evidence for the overexpression of miR-942 as an independent indicator for poor prognosis and a novel therapeutic target for cancer treatment [20]. In patients with OS, there are also some members of miRNA aberrantly expressed during the tumor development. For instance, Hu et al. reported that the expression of miR-1285-3p in OS tissues and cell lines was downregulated and associated with patients' overall survival [21]. Xue et al. focused on the role of miR-638 in OS progression and demonstrated that miR-638 could enhance OS cell apoptosis and inhibit cell proliferation and invasion [22]. miR-1826 has been studied in some human cancers, such as melanoma [14], colorectal carcinoma [13], renal cancer [15], and bladder cancer [23]. The expression patterns of miR-1826 present a contradictory situation that the increased miR-1826 expression was found in colorectal carcinoma, while the decreased miR-1826 expression was observed in melanoma, renal cancer, and bladder cancer. Thus, it is indicated that the role of miR-1826 may vary depending on the types of tumor. In OS patients, this study firstly gave data for the expression of miR-1826 in OS tissues, which showed a decrease in the expression of miR-1826 in OS tissues compared with the adjacent normal tissues. The similar downregulated miR-1826 expression was also found in four OS cell lines, which confirmed our tissue expression data. Moreover, the reduced miR-1826 expression was found to be associated with patients' clinical stage and distant metastasis, and a significant decrease in miR-1826 expression was observed in patients with advanced clinical stage compared with those with early stages, indicating that miR-1826 might be involved in the development of OS.

In recent decades, the clinical significance of miRNAs has received increasing attention on their diagnostic and prognostic value of human cancers [24]. Despite great processes have been made in OS treatment, the prognosis in patients suffering from this malignancy remains dismal. Novel noninvasive prognostic methods are necessary to predict the prognosis of OS. Some miRNAs with abnormal expression have been identified as prognostic biomarkers in patients with OS. For example, the decreased expression of miR-99b in OS tissues was demonstrated as an indicator of poor prognosis for OS patients [25]. miR-93 served as an oncogene of OS and was associated with the overall survival of OS patients [26]. Chen et al. reported that miR-191-5p was upregulated in OS tissues and associated with poor prognosis of OS patients [27]. For miR-1826, its aberrant expression has also been described as an indicator for the overall survival of colorectal carcinoma [13] and renal cancer [15]. According to the survival analysis in this study, the OS patients with low miR-1826 expression had a shorter survival time, and the downregulation of miR-1826 was determined as an independent prognostic indicator for the overall survival in OS patients, indicating that miR-1826 might serve as a novel biomarker for the prognosis of OS.

The downregulated miR-1826 in renal cancer [15] and bladder cancer [23] has been determined as a tumor suppressor by inhibiting tumor cell proliferation, migration, and invasion. Considering the downregulation of miR-1826 in OS tissues, we considered that miR-1826 might also act as a tumor suppressor during OS pathogenesis. By the gain- and loss-of-function analyses in the present study, we found that the OS cell proliferation, migration, and invasion were suppressed by the overexpression of miR-1826, while they were promoted by the silence of miR-1826, indicating the suppressor role of miR-1826 in the progression of OS. However, the mechanisms underlying the role of miR-1826 in OS progression were not assessed, which is a limitation of this study and needs to be systemically studied in further investigations. As a tumor suppressor in bladder and renal cancers, VEGFC, CTNNB1, and MAP2K1 were three targets of miR-1826 to mediate its inhibiting effect on tumorigenesis [15, 23]. Thus, these three genes should be focused in the studies regarding the related molecular mechanisms of miR-1826 acting in OS.

Taken together, miR-1826 is downregulated in OS tissues and cells and serves as an independent prognostic biomarker for OS patients. miR-1826 suppresses OS cell proliferation, migration, and invasion, indicating its tumor suppressor role in OS progression. This newly identified miR-1826 may provide novel insight into the pathogenesis of OS and provide a promising prognostic biomarker and therapeutic target for the treatment of OS.

## Figures and Tables

**Table 1:** Relationship between miR-1826 and the clinical characteristics of the OS patients.

Features	Total no. *N* = 122	miR-1826 expression		*P* values
		Low (*n* = 69)	High (*n* = 53)	
Age (years)				0.780
≤18	65	36	29	
>18	57	33	24	
Gender				0.993
Female	53	30	23	
Male	69	39	30	
Tumor size (cm)				0.626
≤8	66	36	30	
>8	56	33	23	
Clinical stage				0.022
IA-IIA	57	26	31	
IIB-III	65	43	22	
Distant metastasis				0.026
Negative	69	33	36	
Positive	53	36	17	

**Table 2:** Cox regression analysis for the patients with OS.

Variables	Univariate analysis			Multivariate analysis		
	HR	95% CI	*P* value	HR	95% CI	*P* value
miR-1826	2.721	1.499-4.940	0.001	2.452	1.248-4.373	0.014
Age	1.185	0.686-2.047	0.543	1.258	0.724-2.187	0.416
Gender	1.063	0.621-1.821	0.824	1.007	0.583-1.739	0.979
Tumor size	1.045	0.612-1.785	0.871	1.160	0.669-2.010	0.597
Clinical stage	2.253	1.279-3.971	0.005	2.129	1.106-4.099	0.024
Distant metastasis	2.133	1.159-3.925	0.015	1.829	1.007-3.725	0.046

## Data Availability

All the data in this study were included in the manuscript.
